# P02.171. Lessons learned from a preliminary study of Whole Food Diet used by primary care patients

**DOI:** 10.1186/1472-6882-12-S1-P227

**Published:** 2012-06-12

**Authors:** Y Zhang, K Chauncey, Q Gan, M Ragain

**Affiliations:** 1 Texas Tech University Health Sciences Center, Lubbock, USA; 2 Texas Tech University, Lubbock, USA

## Purpose

To address the low dietary adherence issue in clinical practice, it is important to promote a diet plan that has a broad spectrum of diet options to better match individual patient food preferences, lifestyles, and personal health profiles. Whole Foods Diet (WFD) offers more diet options to patients and guides them on how to make the right diet choices. This preliminary study assessed patients’ adherence to the WFD and summarized the lessons learned.

## Methods

A two-site non-randomized clinical trial was conducted among primary care patients. Physicians prescribed WFD to the patients at the intervention site and suggested the patients at the comparison site follow a dietary plan other than WFD. Measures included self-reported dietary adherence rates, waist to hip ratio (WHR), BMI, and eating behaviors measured by the Dutch Eating Behavior Questionnaire (DEBQ). Data collected at baseline, month 1, 2, 6 and 12 were analyzed using individual growth curve (IGC) models with the SAS Mixed Procedure.

## Results

We encountered much unexpected hardship in recruiting the comparison group participants. We enrolled 29 participants in WFD and 14 in comparison group at baseline. There was no statistical difference in participant profiles between the two groups at baseline. Of them, 14 in WFD and 10 in the comparison group remained at month 6, while 7 and 6 remained at month 12, respectively. No difference of adherence rates was found between the WFD and comparison groups. Both restrained and external DEBQ subscale scores increased significantly from baseline to month 6 in the WFD group only. IGC models reveal that WFD participants’ WHRs are declining.

## Conclusion

WFD may help the patients be more cautious about nutrition and caloric balance, but loss to follow-up/adherence is the major issue of this diet study. Our finding suggests that a 6^th^ month diet refesher or re-intervention may be needed for better compliance.

